# Special Issue: Role of Bacterial Chromatin in Environmental Sensing, Adaptation and Evolution

**DOI:** 10.3390/microorganisms9112406

**Published:** 2021-11-22

**Authors:** Remus T. Dame

**Affiliations:** 1 Department of Macromolecular Biochemistry, Leiden Institute of Chemistry, Leiden University, 2333 CC Leiden, The Netherlands; rtdame@chem.leidenuniv.nl; 2 Centre for Microbial Cell Biology, Leiden University, 2333 CC Leiden, The Netherlands; 3 Centre for Interdisciplinary Genome Research, Leiden University, 2333 CC Leiden, The Netherlands

A typical bacterial cell is micron-sized and contains a genome several million base pairs in length. Bacterial genomes are organized and compacted into a structure called the nucleoid by a multitude of factors that include architectural proteins, DNA topology and macromolecular crowding [1,2,3]. Due to interplay between genome organization and DNA transactions, these factors play specific and generic roles in processes such as transcription, replication, repair and chromosome segregation [4,5,6]. Environmental signals altering genome organization thus drive adaptive responses to changes in environment [7,8,9,10]. Additionally, it has become clear that genomic incorporation and maintenance of foreign DNA is facilitated by factors involved in silencing expression of foreign DNA (xenogeneic silencing) until relieved by specific signals [11]. In that light, spatio-temporal organization of the genome is a topic of large current interest, explored at both cellular and molecular levels. Concurrently, in recent years, there has been increasing interest in genome organization in non-canonical model organisms, providing comparative perspectives, demonstrating unanticipated activities of proteins involved in shaping the nucleoid of these organisms and revealing novel architectural proteins [12,13]. In this Special Issue of *Microorganisms*, a number of contributions concerning diverse aspects of bacterial genome organization have been collected. These contributions, both review and research articles, report on the interplay between genome organization and DNA transactions, with a focus on the role of the cell’s environment, in diverse bacterial species.

The nucleoid is hierarchically structured at multiple length scales [2,14,15,16,17]. At the nm scale, the genome is structured due to the binding of small architectural proteins that act to bend or wrap DNA or to facilitate DNA–DNA interaction [18,19,20,21]. Genomic DNA with associated proteins, imposing structure and organization, is also referred to as bacterial chromatin [18,22,23]. Many of these proteins have been shown to play specific regulatory roles in DNA transactions, specifically in regulating transcription, as activators or repressors [2,24,25,26]. These proteins are generally referred to as nucleoid-associated proteins (NAPs), bacterial chromatin proteins or, in older literature, histone-like proteins. Higher levels of organization consist of separation into structural and functional domains. So called topological domains are on average about 10 thousand base pairs in size [27,28]. Chromosomal Interaction Domains (CIDs) [29] and macrodomains [30] are, respectively, one or two orders of magnitude larger in size. It is not fully clear what the structural determinants of these higher order structures are: roles have been implied for transcription, DNA supercoiling and specific proteins involved in chromatin organization [1,14,15]. It has also been suggested that the global structure (and therewith also the position of the nucleoid within the cell) is determined by anchoring of the genome to the membrane [31,32,33]. In this Special Issue, Joyeux investigates this hypothesis by performing simulations in which he assumes that formation of the nucleoid results from a physico-chemical process referred to as phase separation, which is a consequence of macromolecular crowding [34,35]. His simulations suggest that, in the absence of any other positioning mechanism, in cells with elongated shapes such as *E. coli*, the nucleoid would be localized at one of the poles. His simulations underline the notion that mechanisms in cells are in place that aid in positioning either by direct membrane anchoring or (dynamic) association of large subcellular structures [36]. Wien et al., on the other hand, report studies on the structural properties of the nucleoid-associated protein Hfq. The authors build further upon earlier observations that this protein is able to self-assemble and to bridge DNA [37,38]. Here, using various spectroscopic methods the authors provide evidence for close DNA packing in structures assembled in alignment with amyloid-like Hfq protein filaments [39]. Such DNA alignment can have functional importance in processes such as genomic integration of foreign DNA or DNA repair [40,41]. In a related study, Parekh et al. investigate the possible role of Hfq in genome evolution. These authors investigate the structure and function of DNA repeat motifs, specifically a G-quadruplex forming sequence, and the interaction of Hfq with quadruplex DNA. The authors show that during transcription G-quadruplex DNA is formed at such sequences in *E. coli* and that such structures promote genetic instability. In the absence of Hfq the mutation rates at these sequences is reduced, suggesting an involvement of Hfq. Indeed, the authors show that Hfq interacts specifically with G-quadruplexes and suggest that the protein by stabilizing its structure yields stronger roadblock activity during replication, increasing mutagenesis [42].

Next to the research articles summarized above, this Special Issue hosts a number of reviews on important aspects of bacterial genome organization, genome activity and genome stability. It has become evident that genome structure and DNA transactions are tightly interwoven. Muskhelishvili et al. discuss in their review the existence and nature of so-called coherent domains of transcription (CODOs), which can be up to several hundreds of thousands of basepairs in size [43]. Specifically, the authors focus on discussing the differences between the commensal bacterium *E. coli* and the plant pathogen *D. dadantii.* The differences in genome organization between these organisms are proposed to reflect specific demands on gene expression in pathogenic bacteria required for environmental adaptation [44]. Kawalek et al. review the many roles of the parB protein, which is, besides its primary roles in DNA segregation, chromosome compaction, intracellular positioning and SMC loading, involved in controlling chromosome replication initiation, cell division and gene regulation. Of particular interest is that the authors highlight inter-species differences in the function of parB [45]. Morikawa et al. review the effects of oxidative stress such as encountered by pathogens when phagocytosed. The authors focus specifically on changes in the ultrastructure of the nucleoid and gene expression of opportunistic human pathogen *S. aureus* and point out key differences with *E. coli* [46]. Finally, Kivisaar reviews evidence that bacterial chromatin structure correlates with differences in mutation and recombination rates across the genome, for instance following exposure to osmotic stress [47]. Such observations have been reported for diverse bacterial species, and specific correlations with genomic binding patterns of NAPs have been established [48,49]. As a consequence of differences in structure and genome accessibility the rate of genome evolution due to mutations and recombination may differ between different genomic regions.

The articles in this Special Issue highlight the complexity of bacterial chromatin and the need for a holistic approach in which structural and functional information at different length and time scales is integrated. Whereas fundamental principles in relation to bacterial chromatin structure and function must be conserved, a picture of large diversity in precise mechanisms and functions across bacterial species is emerging. Above all, the diversity of experimental and theoretical approaches and strategies discussed in the articles in this issue underline the importance of an approach that extends across disciplinary boundaries [16,17,50].
